# *Tripterygium wilfordii Hook. f.* Preparations for Rheumatoid Arthritis: An Overview of Systematic Reviews

**DOI:** 10.1155/2022/3151936

**Published:** 2022-04-12

**Authors:** Huimin Li, Ruixue Hu, Simin Xu, Zeqi Dai, Xue Wu, Jing Hu, Xing Liao

**Affiliations:** ^1^Centre for Evidence-Based Chinese Medicine, Institute of Basic Research in Clinical Medicine, China Academy of Chinese Medical Sciences, Beijing, China; ^2^Department of Clinical Epidemiology, Beijing Traditional Chinese Medicine Hospital, Capital Medical University, Beijing Institute of Traditional Chinese Medicine, Beijing 100010, China

## Abstract

**Objectives:**

To summarize the quantity and quality of evidence for using *Tripterygium wilfordii Hook. f.* (TwHF) preparations in patients with rheumatoid arthritis (RA) and to find the reasons of the disparity by comprehensively appraising the related systematic reviews (SRs).

**Methods:**

We performed an overview of evidence for the effectiveness and safety of TwHF preparations for patients with RA. We searched seven literature databases from inception to July 15, 2021. We included SRs of TwHF preparations in the treatment of RA. Four tools were used to evaluate the reporting quality, methodological quality, risk of bias, and the certainty of evidence for the included SRs, which are the PRISMA, the AMSTAR-2, the ROBIS, and the GRADE approach.

**Results:**

We included 27 SRs (with 385 studies and 33,888 participants) for this overview. The AMSTAR-2 showed that 19 SRs had critically low methodological quality and the remaining 8 had low methodological quality. The rate of overlaps was 68.31% (263/385), and the CCA (corrected covered area) was 0.53, which indicated the degree of overlap is slight. Based on the assessment of ROBIS, all 27 SRs were rated as low risk in phase 1; one SR was rated as low risk in domain 1, 9 SRs were in low risk in domain 2, 16 SRs were in low risk in domain 3, and 16 SRs were in low risk in domain 4 in phase 2; 7 SRs were rated as low risk in phase 3. Among 27 items of PRISMA, 15 items were reported over 70% of compliance, the reporting quality of 16 SRs was rated as “fair,” and 11 were “good.” Using GRADE assessment, moderate quality of evidence was found in 5 outcomes, and 5 outcomes were low quality.

**Conclusion:**

The use of TwHF preparations for the treatment of RA may be clinically effective according to the moderate-quality evidence. There are methodological issues, risk of bias, and reporting deficiencies still needed to be improved. SRs with good quality and further randomized clinical trials that focus on clinical important outcomes are needed.

## 1. Introduction

Rheumatoid arthritis (RA) is the most common autoimmune inflammatory arthritis in adults with a prevalence of 0.5–1.0% of the general population [1, 2]. A recent meta-analysis found the global prevalence of RA was 460 per 100,000 population in the period 1980 to 2019 [3]. RA is characterized by progressive symmetric arthritis with chronic joint inflammation, synovial hyperplasia, and systemic manifestations [4]. The most common symptoms reported by people with RA are arthralgia, swelling, redness, and limited motion range [5, 6]. Without adequate treatment, RA can lead to severe joint deformity and disability, impacting upon patients' quality of life and work ability [7]. Complications associated with RA lead to high morbidity and rising mortality [7, 8]. Significant progress in studying the mechanisms of RA has been made in the field of genetic predisposition and environmental research area, which were involved in its onset and progression, emphasizing the heterogeneity of RA [9].

Treatment algorithms for patients with RA involve measuring disease activity with composite indices, and its treatment target is the maintenance of remission/low disease activity or prevention of joint destruction and deformity and improvement of joint function [10]. The 2021 American College of Rheumatology Guideline for the Treatment of Rheumatoid Arthritis addressed the treatment for patients with RA is the disease-modifying antirheumatic drugs (DMARDs) [11]. As the first line of therapy for RA, (cs)DMARDs (e.g., methotrexate (MTX), leflunomide, and sulfasalazine) and several recommendations against the use of glucocorticoid therapy are made in the newest guideline [12]. Although the prospects for most patients are now favorable, many still do not respond to current therapies. Adverse effects (e.g., immunosuppression, bone marrow dysfunction, interstitial lung disease, liver damage, hyperglycemia, and hypertension) occurred in RA patients with longtime medication given for treatment [13, 14], and the cost of treating RA has also risen strikingly, largely as a consequence of the biologic therapies [15]. Accordingly, there are still some unmet needs for patients who do not achieve remission and who continue to worsen despite treatment. Hence, patients often seek more complementary therapies.

The popularity of complementary and alternative medicine (CAM) in the management of RA has grown considerably, which covered both the interest of patients and the research community over the past decade [16, 17]. Botanical extract, among the CAM approaches, is an effective option against RA symptoms owing to several anti-inflammatory, palliative, and antiarthritic properties. *Tripterygium wilfordii Hook. f.* (TwHF) is a traditional Chinese herb, which is widely used in the treatment of RA in China [18, 19] due to its anti-inflammatory and immunosuppressive effects. Several TwHF preparations and patented preparations derived from TwHF extracts are clinically available, including Tripterygium wilfordii tablets (TWTs) and Tripterygium wilfordii glycosides tablets (TWGTs) and Tripterygium hypoglaucum hutch tablets. Both TWTs and TWGTs exhibited efficacy similar to MTX as well as enhanced efficacy when a combined remedy of the tablets and MTX was administered to patients with RA in randomized controlled clinical trials [20, 21]. Biochemical and pharmacokinetic studies found that triptolide (TP) and celastrol are two of the most bioactive, yet toxic, constituents identified in TwHF preparations [22]. Triptolide is regarded as the most potent systematic anti-inflammatory and immunoregulating natural products [23]. Previous reviews summarized that the mechanisms associated with the significant therapeutic effects of TP and celastrol against T helper cell-mediated immunity, including RA, have been extensively studied [24, 25]. Emerging evidence suggests that TP suppresses inflammatory responses by attenuating MAPK/NF-*κ*B activation and inhibiting downstream responses [24, 25]. Several studies have demonstrated that TwHF preparations' therapeutic effect may be dependent on the immune balance of Th17 cells and Tregs, the regulation of the proportion between CD4+ and CD8+ T cells, and the differentiation of dendritic cells [26, 27]. A large number of individual trials and systematic reviews (SRs)/meta-analyses (MAs) of TwHF preparations in the treatment of RA have been published. However, the results and quality of the SRs have been mixed. As an increasingly popular form of evidence synthesis, an overview of SRs/MAs uses explicit and systematic methods to extract and analyze their results across important outcomes from multiple SRs/MAs on related research questions [28, 29]. Thus, we conducted an overview of SRs/MAs about TwHF preparations in the treatment of RA to inform healthcare decision-makers and address new questions that were not reported in the included SRs/MAs.

## 2. Methods

We adhered to two guidelines for conducting an overview, one is the Cochrane Handbook [30], and the other is the Preferred Reporting Items for Systematic Reviews and Meta-Analyses (PRISMA) [31]. Literature search and selection, data extraction, and quality evaluation were completed by two authors (Huimin Li and Simin Xu) independently. All discrepancies were resolved by consulting an experienced third reviewer (Xing Liao) firstly and then reached consensus in the team of all authors. We referred to two published overviews with good quality in this step [32, 33].

### 2.1. Protocol and Registration

We registered our protocol in the International Platform of Registered Systematic Review and Meta-Analysis Protocols (DOI number is 10.37766/inplasy2021.8.0081). There was no need for the ethical approval.

### 2.2. Search Strategy

We searched the following literature databases using the words of TwHF, RA, and systematic review/meta-analysis from inception to July 13, 2021: The Cochrane Library, PubMed, Embase, VIP database, China National Knowledge Infrastructure, CBM, and WanFang. The details of the literature search strategy are presented in Appendix A.

### 2.3. Inclusion and Exclusion Criteria

We selected related SRs meeting inclusion criteria: (a) SRs of randomized controlled trials (RCTs) or other research designs; (b) the participants were diagnosed as RA by common criteria, or it was clearly stated that the population of the SR was RA patients; (c) the comparisons were any type of TWHs extract with or without standardization treatment used as the treatment for RA versus standardization treatments, such as drug therapy, routine activities, no therapy, placebo, and other treatment; (d) outcomes including clinical, physiological, or caregiver-reported outcomes; patient-reported outcomes; and adverse effects. Only SRs published in English and Chinese were included. SRs with unavailable full text were excluded.

### 2.4. Data Management and Data Collection

We used the literature manager NoteExpress (V3.5.0.9054) to perform literature selection. We firstly screened title and abstract to eliminate duplication for potentially relevant SRs. Full texts of possible eligible SRs were downloaded and assessed based on inclusion and exclusion criteria. We applied a predesigned form to extract related information from each eligible SR: general information (e.g., the publication year, title, first author, country, and language); review characteristics (e.g., literature database, number of included studies and participants, diagnosis criteria, interventions and comparisons, meta-analysis, quality assessment tool, and outcomes); and the main conclusion.

### 2.5. Assessment of Methodological Quality

We used the tool Assessing the Methodological Quality of Systematic Reviews 2 (AMSTAR-2) [34] to estimate the methodological quality for all included SRs, which provides guidance to rate the overall confidence in the results of a review. The AMSTAR-2 includes four critical domains, which are preparation for review, search for and selection of primary studies, data coding and reporting, and data synthesis. It contains 16 items, of which seven were critical domains (items: 2, 4, 7, 9, 11, 13, and 15) that can critically influence the validity of an SR and its conclusion. For each item, three options could be chosen to answer the question: “yes” indicating high quality, “partial yes” being partially compliant, or “no” being poor quality. The overall rating depends on weaknesses in the critical domains (items: 2, 4, 7, 9, 11, 13, and 15). The rating is divided into four categories depending on the number of critical flaws and/or noncritical weaknesses: “high” means no or one noncritical weakness; “moderate” means more than one noncritical weakness but no critical flaws; “low” means one critical flaw with or without noncritical weaknesses; and “critically low” means more than one critical flaw with or without noncritical weaknesses.

### 2.6. Assessment of Risk of Bias

We also evaluated the risk of bias of each included SR/MA using ROBIS statement [35], which assesses whether an SR is at risk of bias based on its methods and conduct. ROBIS is comprised of three phases: (a) assess relevance (optional), (b) identify concerns with the SR process, and (c) judge risk of bias of the SR. Phase one is optional, which assesses the relevance. Phase two includes four domains formed by 21 signaling questions, which aims to identify concerns with the review process. Phase three, with three signaling questions, concentrates to judge the risk of bias of the SR. All signaling questions were answered as “yes,” “probably yes,” “probably no,” “no,” and “no information.” Based on the answers to the signaling questions in each domain, each domain is assigned a risk of bias grade. If all of signaling questions of phase 3 were answered as “yes,” the SR was judged as “low risk.” Any of signaling question of phase 3 was answered as “probably no” or “no,” the SR was assessed as “high risk.” If the information provided was insufficient to judge, the SR was rated as “unclear risk.” After completing phase three, a summary judgment (e.g., high, low, or unclear) regarding the risk of bias for the SR will be rendered.

### 2.7. Assessment of Reporting Quality

We applied the checklist Preferred Reporting Items for Systematic Reviews and Meta-Analyses (PRISMA) [36] to appraise the report quality for each SR/MA. PRISMA consists of seven main domains: title, abstract, introduction, methods, results, discussion, and funding. It comprises 27 items and a four-phase flow diagram, which focus on the reporting of methods and results in SRs/MAs. Each item was answered as “yes,” “no,” and “partially reported.” With the purposes of statistical analysis, we judged whether an SR fully reported what was required by PRISMA and scored each item with a 1 point (fully reported), 0.5 point (partially reported), or 0 point (not reported) for each item. The sum of all items scored for each question was divided by its maximum possible score as a percentage to assess the report quality for each SR. The report quality of SRs related to its PRISMA score percentage was rated as very poor (<30%), poor (30–50%), fair (50–70%), good (70–90%), and excellent (>90%).

### 2.8. Assessment of Quality of Evidence

The Grades of Recommendations, Assessment, Development, and Evaluation (GRADE) [37] approach was used to assess and report the certainty of evidence for the clinically important outcome of interest in the current overview. In the GRADE system, five factors for rating down the quality of evidence were considered for the current overview: risk of bias (also called “study limitations”), inconsistencies, indirectness, inaccuracy, and publication bias. Quality of evidence of each outcome was judged as “high,” “moderate,” “low,” and “very low.”

### 2.9. Data Synthesis and Presentation

We narratively described the characteristics of included SRs and the efficacy and safety of TwHF preparations for RA in this overview. We made use of tabulation and figures to summarize the results of all SRs/MAs as well as the appraisal results from AMSTAR-2, PRISMA, and ROBIS. We generated the evidence profile and summary of findings table with the aid of the GRADEpro GDT online software (https://www.gradeworkinggroup.org/).

## 3. Results

### 3.1. Results on SRs/MAs Search and Selection

**Figure 1:**
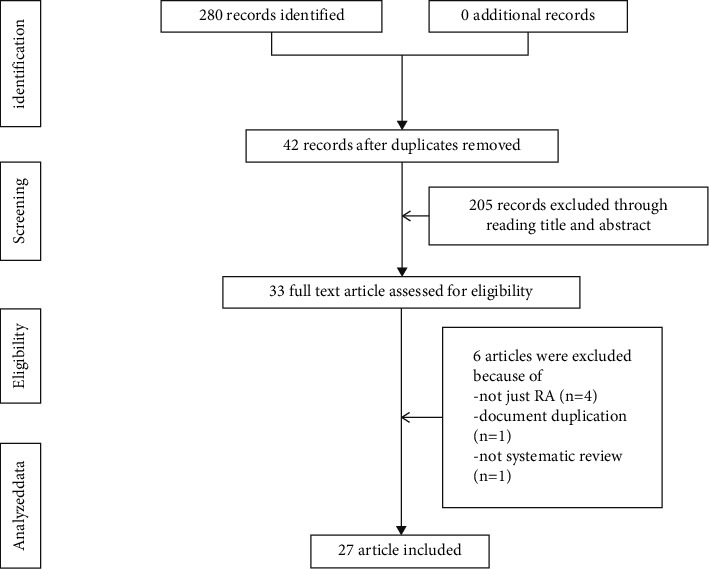
Flow chart showing the selection of SRs from search to inclusion.

The initial search strategy yielded 280 records from the selected databases. After removal of 42 duplicates, 238 records were screened based on title and abstract. Afterward, fifty-six articles were read in full text, of which 27 SRs [20, 21, 38–62] were included in the current overview. The excluded review list has been recorded in Appendix A. The PRISMA diagram for the process of screening and selecting SRs is displayed in Figure 1.

### 3.2. Characteristics of Included Reviews

Of the 27 included SRs, 19 [38, 40–44, 46, 47, 50–54, 57–62] were published in Chinese and 8 [20, 21, 39, 45, 48, 49, 55, 56] in English. They were published from 2013 to 2021, including 26 SRs from China and 1 from the United Kingdom. All 27 SRs included RCTs, of which 26 conducted meta-analysis, while only one SR [39] did qualitative analysis; of which 25 SRs evaluated efficacy and safety of TwHF preparations, while the remaining 2 [58, 60] only explored the safety profile of TwHF preparations; and of which 24 SRs only included RCTs, while the remaining 3 included mixed studies. The number of studies included in each SR varied from 2 to 79, and sample sizes of individual study ranged from 105 to 5255. Among the 27 SRs, 20 SRs [20, 21, 40–47, 49, 51, 53–57, 59, 61, 62] specified the diagnostic criteria of the included studies, while the remaining seven [38, 39, 48, 50, 52, 58, 60] were unclear. As for intervention, 13 SRs [20, 38–46, 54, 60, 62] were TwHF preparations plus other treatment (e.g., routine drug therapy or placebo) versus other treatment alone, and 14 SRs [21, 47–53, 55–59, 61] were TwHF preparations versus other treatments (e.g., routine drug therapy). The outcomes reported by the 27 SRs covered tender joint count (TJC), swollen joint count (SJC), morning stiffness (MS), grip strength (GS), erythrocyte sedimentation rate (ESR), C-reactive protein (CRP), rheumatoid factor (RF), American College of Rheumatology (ACR), adverse events (AEs), interleukin 1 (IL-1), interleukin 4 (IL-4), interleukin 6 (IL-6), interleukin 10 (IL-10), tumor necrosis factor-alpha (TNF-*α*), 15-m walking time (15Mwt), 15/20-m walking time (15/20Mwt), tenderness score, physician-rated and patient-rated overall assessments, X-ray score, radiological changes of joints, withdrawal rate related to adverse reactions, joint symptoms, disease activity score, cyclic citrullinated peptide (CCP), mean grip strength, analgesic onset time (AOT), short form 36 health questionnaire (SF-36), health assessment questionnaire, traditional Chinese medicine symptom score of the joint swelling, and painful joint count. Among the 25 SRs that aimed to evaluate both efficacy and safety of TwHF preparations, only 12 SRs [20, 40–45, 49, 50, 53, 55, 56] reported AEs. The quality assessment tools of the original studies varied among the 27 SRs, out of which 17 employed Cochrane risk of bias tool, 9 adopted the Jadad score, and the remaining 1 used an unknown tool. Out of the 27 SRs, 26 SRs [20, 21, 38, 40–62] completed subgroup analysis, and 6 [47, 51, 54, 55, 57, 61] conducted sensitivity analysis. Of the 27 SRs, 12 SRs [41, 44, 46–48, 50, 52, 54, 55, 57, 59, 61] concluded TwHFPs were probably beneficial, 11 SRs [20, 40, 42, 43, 45, 48, 49, 51, 53, 56, 62] were beneficial, 3 [38, 58, 60] were no effect and 1 [39]) was harmful. The detailed characteristics the SRs are presented in Table 1.

### 3.3. Results on Review Quality Assessment

#### 3.3.1. Methodological Quality

Table 2 presents the results of methodological quality of the 27 included SRs/MAs assessed by the AMSTAR-2. Out of the 27 included SRs, the quality of 20 SRs was rated critically low since they had more than one critical weakness (items 2, 4, 7, 9, 11, 13, and 15). Severe limitation existed in item 2, item 3, item 7, item 10, and item 16 (percentage of items with “yes” < 50%). The methodological quality appraised by the AMSTAR-2 for the 27 SRs can be reflected as follows: 92.6% of the 27 SRs did not explicitly report the review methods, which should be established before conducting the review and significant deviations from the protocol was found (item 2); 91.49% did not provide a list of excluded studies and justified the exclusions (item 7); 96.3% did not explain the selection of the study designs for inclusion in the review (item 3); 81.49% did not use a comprehensive literature search strategy (item 4), 66.67% did not report any potential sources of conflicts of interest (item 16); and 66.67% described the included studies insufficiently (item 8).

#### 3.3.2. Risk of Bias

The ROBIS was used to assess the risk of bias for each SR, the results of which are presented in Appendix B. All 27 SRs were judged with low risk of bias in phase 1 (assessing relevance). Regarding phase 2, across all 27 SRs, the individual bias domains at the highest risk of bias were domains 1 (protocol and eligibility criteria, 26/27, 96.30%) and 2 (methods to identify and select studies, 18/27, 66.67%). Specific areas of concern in these two domains were the lack of information about publication of an SR protocol, language restrictions, choice of literature databases, and searches for gray literature. Eleven (40.74%) SRs were at high risk of bias for both domain 3 (collection and study appraisal) and domain 4 (synthesis and findings). Seven (25.92%) SRs were rated as low risk of bias in phase 3(risk of bias in the review). Finally, 20 of the 27 SRs were rated as “high risk,” and the remaining 7 SRs were rated as “low risk.” In general, 20 of the 27 SRs were rated as “high risk,” and the remaining 7 SRs were rated as “low risk.” Reviews with high risk of bias mainly have problems with the completeness of the search for relevant studies, inadequate report of the protocol, and lack of explicit method to select studies.

#### 3.3.3. Reporting Quality

The results of PRISMA assessment are presented in Appendix B. Of the 27 items, 12 items had adherence greater than 70% in most of the included SRs; however, five items had only one SR, and four items had no adherence. The section of rationale, objectives, eligibility criteria, title, introduction, study characteristics, and results of individual studies were all well reported by all included SRs, but there were still inadequate reports in other sections. Five items with adherence lower than 5% were the main reporting deficiency, which are if a protocol exists or is registered (item 5, percentage of items with “yes,” 3.7%); certainty assessment (item 15, percentage of items with “yes,” 3.7%); search strategy (item 7, yes = 3.7%); structured summary (item 2, yes = 3.7%); and certainty assessment (item 22, yes = 0%). Additionally, only one SR [45] mentioned the study protocol and the protocol registration number. Finally, the reporting quality of 16 SRs was rated as “fair,” and 11 “good.”

#### 3.3.4. Evidence Quality of Outcomes

The information about the efficacy and safety of TwHF preparations for RA from included SRs is summarized and displayed in Table 3. Ten of the 27 SRs that selected rheumatoid factor as the primary outcome suggested that patients with RA who received TwHF preparations had better effects than their counterparts who were treated with DMARDs. Eighteen of the 27 SRs (66.66%) reported that both tender joint count and swollen joint count were significantly reduced in the TwHF preparations group. As for the ACR (20/50/70), 7 of the 27 SRs (25.92%) reported that ACR (20/50/70) was significantly improved in the TwHF preparations group. As for the levels of ESR and CRP, 18 of the 27 SRs (66.66%) reported that both of them were significantly reduced following the TwHF preparations treatment, while one SR reported there was no statistical significance for ESR. Among the 15 included SRs that reported morning stiffness (MS), 8 SRs reported that MS was significantly reduced in the TwHF preparations group. The combination therapy with TwHF preparations and other treatment significantly decreased the duration of morning stiffness; alleviated tender joint count; relieved swollen joint count, ACR (20/50/70), ESR, CRP, and RF; and lowered the level of TNF-*α*. The most common AEs with TwHF preparations were gastrointestinal discomfort, menstruation disorders, amenorrhea, decreased sperm motility, liver function damage, and skin diseases.

*(1) Swollen Joint Count*. Nine MAs [42, 44, 47–49, 53, 54, 59, 60] reported the swollen joint count. Two interventions (TwHFPs with LEF and TwHFPs with MTX) reduced swollen joint count. The result of different comparisons were shown as follows: TwHFPs vs NM (MD: −4.13, 95% CI: −5.69, −2.58; low quality); TwHFPs + LEF vs LEF + CWM + COP (MD: −1.24, 95% CI: −1.59,−0.88; moderate quality); TwHFPs or TwHFPs + DMARDs vs NM (MD: −1.92, 95% CI: −3.85, 0.03; low quality); TwHFPs + LEF vs CWM + COP(MD: 0, 95% CI: −0.19, 0.2; low quality); TGT + MTX vs MTX(MD: 3.01, 95% CI: 2.09, 3.93; moderate quality); TwHFPs vs LEF (SMD: −0.64, 95% CI: −1.32, 0.05; moderate quality); TwHFPs vs CWM + COP (MD: −1.96, 95% CI: −3.56, 0.35; moderate quality); TwHFPs + MTX vs MTX (SMD: −1.46, 95% CI: −2.4, −0.44; moderate quality); TwHFPs + LEF vs LEF (SMD: −0.78, 95% CI: −1.52, −0.04; moderate quality); TwHFPs + DMARDs vs DMARDs (SMD: −1.72, 95% CI: −2.04, −1.41; low quality).

*(2) Morning Stiffness*. Six MAs [47–49, 51, 54, 59] reported the morning stiffness. Two interventions (TwHFPs with LEF and TwHFPs with MTX) reduced morning stiffness. The result of different comparisons were shown as follows: TwHFPs + LEF vs LEF + CWM + COP (MD: −0.29, 95% CI: −0.42,−0.12; moderate quality); TwHFPs or TwHFPs + DMARDs vs NM (MD: −30.94, 95% CI: −37.85, −24.04; low quality); CWM + COP (MD: −0.32, 95% CI: −0.4, −0.24; low quality); TGT + MTX vs MTX (MD: −18.24, 95% CI: −12.64, 23.84; moderate quality); TwHFPs + MTX vs MTX (SMD: −1.51, 95% CI: −2.31, −0.71; low quality); TwHFPs + LEF vs LEF (SMD: −2.29, 95% CI: −3.36, −1.12; moderate quality).

*(3) Rheumatoid Factor*. Nine MAs [21, 39, 41, 42, 50, 51, 54, 59, 60] reported the rheumatoid factor. Two interventions (TwHFPs and TwHFPs with LEF) reduced the rheumatoid factor. The result of different comparisons were shown as follows: TwHFPs + LEF vs LEF + CWM + COP (MD: −0.29, 95% CI: −0.42, −0.12; moderate quality); TwHFPs or TwHFPs DMARDs vs NM (MD: −30.94, 95% CI: −37.85, −24.04; low quality); CWM + COP (MD: −0.32, 95% CI: −0.4, −0.24; low quality); TGT + MTX vs MTX (MD: −18.24, 95% CI: −12.64, 23.84; moderate quality); TwHFPs + MTX vs MTX (SMD: −1.51, 95% CI: −2.31, −0.71; low quality); TwHFPs + LEF vs LEF (SMD: −2.29, 95% CI: −3.36, −1.12; moderate quality).

*(4) Tender Joint Count*. Eight MAs [44, 47, 49, 51, 53, 54, 59, 60] reported the tender joint count. Three interventions (TwHFPs, TwHFPs with DMARDs, and TwHFPs with LEF) reduced TJC. The result of different comparisons were shown as follow: TwHFPs vs NM (MD: −32.4, 95% CI: −89.76, −24.96; low quality); TwHFPs vs CWM + COP (MD: −5.41, 95% CI: −7.46, −3.37; low quality); TwHFPs + LEF vs LEF + CWM + COP (MD: −50.88, 95% CI: −72.3, 29.45; very low quality); TwHFPs vs CWM + COP (MD: −0.5, 95% CI: −0.81, −0.18; low quality); TwHFPs or TwHFPs + MTX vs MTX (MD: −0.5, 95% CI: −0.81, −0.18; moderate quality); TwHFPs vs NT + CWM + COP (MD: 0.38, 95% CI: −0.42, 1.18; low quality); TwHFPs vs LEF (SMD: −2.23, 95% CI: −3.27, −1.19; moderate quality); TwHFPs + MTX vs MTX (SMD: −1.11, 95% CI: −1.96, −0.26; low quality); TwHFPs + LEF vs LEF (SMD: −2.97, 95% CI: −4.22, −1.72; moderate quality).

*(5) Total Effective Rate*. Eight MAs [21, 46, 48, 49, 51, 53, 59, 60] reported the total effective rate. Three interventions (TwHFPs, TwHFPs with DMARDs, and TwHFPs with LEF) increased the total effective rate. The result of different comparisons were shown as follows: TwHFPs vs CWM + COP (RR: 1.20, 95% CI: 1.13, 1.27; moderate quality); TwHFPs + MTX vs NT + CWM + COP (OR: 1.02, 95% CI: 0.46, 2.28; low quality); TwHFPs + LEF vs CWM + COP (RR: 1.19, 95% CI: 1.02, 1.38; low quality); TwHFPs vs LEF (OR: 3.80, 95% CI: 2.34, 6.16; moderate quality); TwHFPs + MTX vs MTX (RR: 1.23, 95% CI: 1.13, 1.35; low quality); TwHFPs + MTX vs MTX (RR: 1.23, 95% CI: 1.13, 1.35; low quality); TwHFPs + DMARDs vs DMARDs (RR: 1.23, 95% CI: 1.133, 1.335; moderate quality).

#### 3.3.5. Overall Quality of the Evidence

The details of GRADE summary of findings are described in Table 3. We only rated the body of evidence for main outcomes that were pooled based on RCTs using the GRADE system. Nineteen SRs involving 5 main outcomes related to the effects of TwHF preparations for RA were analyzed. Based on the analysis of the GRADE approach, moderate quality of evidence was found in 5 outcomes of the included SRs, whereas 5 outcomes were rated as low quality, and 2 outcomes were very low quality. There was no outcome with high-quality evidence found in the current overview. Risk of bias (*n* = 13) was the most common downgrading factors, followed by inconsistency (*n* = 2), imprecision (*n* = 6), publication bias (*n* = 5), and indirectness (*n* = 9). The reasons to downgrade the level of evidence are the poor methodological quality, imprecision of the results, and small sample size among relevant trials. The downgraded reason the small number of participants was for the majority outcomes. The number of participants included in the SR did not reach the optimal information size. Then, the quality of evidence was downgraded due to its imprecision. The effect estimates could not provide a convincing explanation for differences in results across studies for nearly half of the outcomes, owing to the statistically significant heterogeneity. Some of the outcomes had publication bias because of the incomprehensive literature search, which was already found by AMSTAR-2 and ROBIS.

## 4. Discussion

Overviews are most frequently employed where multiple systematic reviews already exist on similar or related topics and aim to systematically bring together, appraise, and synthesize the results of related systematic reviews [67]. Although there are an increasing number of SRs/MAs published on TwHF preparations for RA, the quality of those SRs/MAs taken together has not been assessed until now. Thus, there is a need to systematically bring together, appraise, and synthesize the results of related systematic reviews in an overview of this issue.

### 4.1. Summary of Main Findings

*Tripterygium wilfordii Hook f.* (TwHF, also known as Thunder God Vine or Lei Gong Teng) is one of the most representative traditional Chinese herbs with therapeutic potential that has been broadly studied by scientists. In spite of some occasional, but severe, adverse effects (which may be harmful to the liver, kidneys, reproductive tissues, and immune tissues [64]) found in clinical practice, the use of TwHF preparations is still not reduced due to their significant efficacy against diseases. In the current overview, 27 included SRs on TwHF preparations were published from 2013 to 2021. Out of the included 27 SRs, 26 of which drew positive conclusions of TwHF preparations for RA; however, none of the review authors drew a firm conclusion owing to the small sample size of the included RCTs or their low methodological quality. Though it showed that adverse events caused by TwHF preparations were not significantly different from those caused by immunosuppressive agents, there is an urgent need for improving prevention and management of patients' tolerance and monitoring the administration of TwHF preparations in the clinical practice [65]. And TwHF preparations should not be used for RA patients with liver and kidney insufficiency and fertility planning, in view of the liver and kidney and reproductive toxicity. We reclassified and examined the 385 primary studies included in the 27 included SRs. We calculated the percentage of primary studies included in more than one SR and the rate of CCA (corrected covered area), which is a measure about the degree of overlap [68]. The rate of overlaps was 68.31% (263/385) and the CCA was 0.53, which indicated the degree of overlap is slight. There are two possible reasons for the overlap, one is SRs in TCM research area often having a broader research question, for instance, the majority of SRs investigating TwHFPs versus conventional medicine on different outcomes, leading to more primary studies included in an SR; the other is some authors of SRs reported that the quality of the published SRs was poor and there was necessity to perform a new one rather than an updated one. The quality of the SRs and the evidence quality of the outcomes in this overview are generally discouraging, on the basis of the evaluation from AMSTAR-2, ROBIS, PRISMA, and GRADE, implying that there is huge disparity between the included SRs/MAs and the real world. Thus, in view of these limitations, the trustworthy of evidence for TwHF preparations for RA was weakened. Consequently, recommending TwHF preparations as a complementary or even alternative treatment for patients with RA should be cautious.

The current overview found four main findings. First of all, the methodological quality of all the SRs was rated as critically low or low by the AMSTAR-2 tool, and the following deficiencies existed: 1) selective reporting bias arose due to the lack of SR protocol or the absent registration of the protocol of the included SRs, which affected their thoroughness; 2) the confidence of results was influenced by the decreased transparency, due to the omission of the lists of excluded studies with explanations; and 3) the reliability of the conclusions and its impact on different users of reviews were affected by missing disclosure of potential financial conflicts of interest or the authors' conflicts of interest. Secondly, high risk of bias evaluated by the ROBIS tool was found in the literature search, study selection, data synthesis method, and the explanation in the discussion among these included SRs, which made the current evidence unreliable. Thirdly, the assessment on included SRs' adherence to the PRISMA statement found that incomplete reporting occurred in the literature search strategies, the literature screening processes, the additional analyses, and the sources of funding, which decreased the trustworthiness of the findings. When information is absent or ambiguous in the reporting, SR users cannot implement the findings of SRs into clinical practice. Lastly, the results from the GRADE assessment in this overview revealed that moderate-quality evidence on some outcomes for TwHF preparations having potential effects for patients with RA. Low-quality evidence affected the confidence in the evidence, which made the uncertainty about the trade-offs when recommending the TwHF preparations as an intervention for RA. In regard to the safety of TwHF preparations for RA, 11 SRs reported that the combined therapy increased clinical efficacy significantly when compared with the Western medicine alone, whereas four SRs found no difference between the two groups. In the current overview, there is no high-quality evidence, and most of the outcomes were rated as low or very low quality. Evidence quality was downgraded due to the study limitations, inconsistency, and the publication biases. The publication bias in most of the included SRs was mostly caused by the small number of included RCTs with small sample size as well as positive results, which may lead to overestimating the effect size.

More than that, most of the original studies of TwHF preparations in treating RA have major limitations, including lack of allocation concealment; subjective outcomes without blinding; loss to follow-up; and no intention to treat analysis, which biased the estimates of the treatment effect and affected the confidence in the estimate of effect in SRs. Study heterogeneity prevented meaningful meta-analysis due to the various evaluation criteria for the assessment of clinical effectiveness and different treatment courses across studies. Only one SR [61] conducted subgroup analysis based on the different treatment courses.

### 4.2. Implications for Future Clinical Practice and Research

According to our results, TwHF preparations may be effective for RA patients, which is consistent with a related previous overview [68]. However, the administration should be monitored due to its adverse effects. TwHF preparations are likely to improve the physical function and quality of life in patients with RA, not just laboratory outcomes. More than half of the included SRs (66%) showed the significant decrease for swollen joint count and tender joint count in the TwHF preparations group, 48.14% for morning stiffness, and only 26% for ACR (20/50/70). But as aforementioned, we should consider the inadequacy of the available evidence and be cautious when recommending TwHF preparations as a treatment for RA patients.

As we all know, the quality of a systematic review depends on the quality of the original research. Therefore, well-designed primary studies should be carried out in the future. The composite outcome total effective rate was used as a primary outcome with a simple rate calculation formula in most studies, whereas relieving joint pain was the internationally considered outcomes. For the sake of producing accepted efficacy evidence of TwHF preparations in the treatment of RA, future studies should select well-recognized outcomes and related measurements that are recommended by expert consensus or by international guidelines [66]. When evaluating the effects of TwHF preparations, we should not only consider the laboratory outcomes and physician-reported outcomes but also take into account patient-reported outcomes (such as quality of life), which can comprehensively evaluate the efficacy of TwHF preparations in the treatment of RA. Additionally, none of the included SRs mentioned follow-up. Considering that RA is a progressive disease with a long disease course, future studies should attach importance to the follow-up period to further assess the long-term efficacy of TwHF preparations for treating RA as well as fully monitoring its toxicity.

Last but not least, we strongly recommend authors of future SRs conduct and report SRs adhering to the AMSTAR-2 tool, ROBIS tool, and PRISMA statement.

### 4.3. Strengths and Limitations

To the best of our knowledge, this is the first systematic overview to explore the evidence of TwHF preparations for RA by using the AMSTAR-2, ROBIS, PRISMA, and GRADE. From the current overview, the quality of the SRs/MAs and body of evidence across outcomes are presented, which may be helpful for the research and clinical practice of TwHF preparations in treating RA. However, there are several limitations in this overview that should be taken into account. We only searched SRs in English and Chinese, which might produce publication bias. Although there are overlapping studies across the included SRs, we did not remove duplicate data and duplicate studies. As we are not aimed to resynthesize the data to evaluate the efficacy of the intervention, the overlap is unlikely to have an impact on the conclusion. The author team members may have their own subjective views during the evaluation, which could result in bias and influenced the research findings. Finally, out of the 27 included SRs, 26 from Chinese researchers supported the use of TwHF preparations for RA, whereas one SR from the British researchers disapproved the use of TwHF preparations, which may be judged as certain ethical bias.

## 5. Conclusion

TwHF preparations may be a complementary and alternative treatment for RA; however, it must be used carefully and monitored for its potentially severe toxicity. The quality of published SRs/MAs is unsatisfactory; hence, further standardized and rigorous SRs/MAs and RCTs are warranted to provide strong evidence for definitive conclusions.

## Figures and Tables

**Table 1:** Characteristics of 27 included systematic reviews.

Author, year	Design and number of included studies	Participants (*n*)	Literature databases	Population diagnostic criteria	Intervention/comparison	Methodological quality assessment tool	Meta analysis (yes/no)	Outcomes	Conclusion
Liu, 2013 [20]	RCT: 10	733	(1)(2)(3)(4)(5)(6)(7)(9)	ACR 1987	TwHFPs vs NM	JS	Yes	TJC; SJC; MS; GS. RF; ESR; CRP; AEs	Beneficial
Wang, 2017 [21]	RCT: 6	643	(6)(7)(8)	ACR	TwHFPs + MTX vs NM	JS	Yes	ACR (20/50); SJC; TJC; MS; ESR; CRP; RF	Probably beneficial
Xu, 2001 [38]	RCT: 3; CCT: 4	784	(4)	NM	TwHFPs vs CWM + COP	CROB	Yes	CTE	No effect
Canter, 2006 [39]	RCT: 2	105	(9)(10)(11)(12)	NM	TwHFPs vs CWM + COP	JS	No	TS; SJC; MS; GS; 15Mwt; ESR; CRP; IgG; IgM; IgA, PPOA	Harmful
Jiang, 2009a [40]	RCT: 8	470	(1)(2)(4)(5)(7)(9)(10)(12)	ACR 1987	TwHFPs vs NT + CWM + COP	CROB	Yes	ACR (20); RF; AEs	Beneficial
Jiang, 2009b [41]	RCT: 7	393	(1)(2)(4)(5)(7)(9)(10)(12)	ACR 1987	TwHFPs vs CWM + COP	CROB	Yes	CTE; X-RS; SJC; ESR; CRP; RF; AEs	Probably beneficial
Tang, 2010 [42]	RCT: 11	2327	(1)(2)(3)(4)	ACR 1987	TwHFPs vs NT + CWM + COP	NA	Yes	ESR; CRP; TJC; SJC; MS; MS; RF; CTE; AEs	Beneficial
Wang, 2011 [43]	RCT: 10	632	(1)(4)(5)(14)	ACR	TwHFPs vs NT + CWM + COP	JS	Yes	ACR (20/50/70) PAOS; VAS; HAQ; CRP; TJC; SJC; ESR; CRP; MS; GS; RCJ; AEs	Beneficial
Wang, 2014 [44]	RCT: 15	1031	(1)(4)(5)(6)(10)(14)	ACR 1987	TwHFPs vs CWM + COP	JS	Yes	TJC; SJC; MS; GS,ESR; CRP; RF; ACR (20/50/70); AEs	Probably beneficial
Wang, 2016 [45]	RCT: 22	5255	(1)(2)(3)(4)(5)(6)(9)	ACR 1987	TwHFPs vs CWM + COP	CROB	Yes	ACR (20/50/70), PPOA; AEs	Beneficial
Yang, 2016 [46]	RCT: 10	889	(1)(3)(4)(5)(6)(9)(10)	ACR 1987	TwHFPs vs CWM + COP	CROB	Yes	TJC; SJC; MS,GS; RF; ESR; CRP	Probably beneficial
Zeng, 2017 [47]	RCT: 6	362	(1)(2)(3)(6)(9)	ACR 1987; ACR/EULAR 2009	TwHFPs + MTX vs NT + CWM + COP	JS	Yes	WDAR; TWR ACR (20/50/70); CRP;CTE; RF; DAS28; SJC; TJC; MS; PPOA; VAS; HAQ; ESR; CRP; EPOTNF-a; IL-10; DAS28	Probably beneficial
Wang, 2018 [48]	RCT: 11	1055	(1)(2)(3)(6)(7)(9)(15)(16)	NM	TwHFPs vs CWM + COP	CROB	Yes	ACR (20/50/70); DAS; ESR; RF; CRP; CCP; hsCRP; TJC; SJC; MGS; 15/20Mwt; AOT; SOT; SF-36; HAQ	Beneficial
Zhou, 2018 [49]	RCT: 14	1254	(1)(3)(4)(5)(6)(9)(10)	ACR 1987	TwHFPs or TwHFPs + DMARDs vs NM	CROB	Yes	TJC; SJC; GS; MS; ESR; CRP; AEs	Beneficial
He, 2018 [50]	RCT: 4	230	(1)(2)(3)(5)(6)(8)	NA	TwHFPs + LEF vs CWM + COP	CROB	Yes	JS; ESR; CRP; CTE; MS; TJC; SJC; AEs	Probably beneficial
Wang, 2019 [51]	RCT: 18	1764	(1)(2)(3)(5)(6)(9)	ACR/EULAR 2010; ACR 1987	TGT + MTX vs MTX	CROB	Yes	TJC; SJC; MS	Beneficial
Li, 2019 [52]	RCT: 25	2507	(1)(2)(3)(5)(6)(9)	NM	TwHFPs or TwHFPs + MTX vs MTX	CROB	Yes	ESP; CRP; RF	Probably beneficial
Yin, 2019 [53]	RCT: 10	792	(1)(2)(3)(4)(6)(9)	ACR/EULAR 2009	TwHFPs + MTX vs MTX	CROB	Yes	CTE; MS; TJC; SJC; TCMSJS; ESP; CRP; RF; AEs	Beneficial
Zhu, 2019 [54]	RCT: 3	233	(1)(2)(3)(4)(5)(6)(7)(8)(9)	ACR1987; ACR/EULAR 2010	TwHFPs vs MTX + SASP	JS	Yes	Mtss; JE; JSN	Probably beneficial
Wen, 2020 [55]	RCT: 40	3092	(1)(2)(3)(4)(5)(6)(8)(9)	ACR 1987; ACR/EULAR 2010	TwHFPs + DMARDs vs DMARDs	CROB	Yes	MS; TJC; SJC; VAS; CRP; ESR; RF; CTE; AEs	Probably beneficial
Yang, 2020 [56]	RCT: 12	830	(1)(2)(3)(6)(9)(10)	ACR/EULAR 2010	TwHFPs + LEF vs LEF + CWM + COP	CROB	Yes	CTE; MS; TJC; SJC; ESR; CRP; RF; IL-1; IL-6; TNF-*α*; AEs	Beneficial
Chen, 2020 [57]	RCT: 10	1184	(1)(2)(3)(4)(6)(9)	ACR 1987; ACR/EULAR 2010	TwHFPs or TwHFPs + MTX vs MTX	CROB	Yes	ACR (20/50/70)	Probably beneficial
Li, 2020 [58]	RCT: 54; CCT: 11; case series: 7; case report: 7	3358	(1)(2)(3)(4)(5)(6)(9)	NM	TwHFPs or TwHFPs + CWM + COP vs NM	CROB; IHE; MIORS; JBI; standard for case report	Yes	AEs	No effect
Wang, 2020 [59]	RCT: 10	876	(1)(2)(3)(4)(5)(6)(7)(8)(9)	ACR 1987; ACR/EULAR 2010	TwHFPs + MTX vs CWM + COP	CROB	Yes	IL-17; IL-23; TNF-*α*; IL-1; IL-6; IL-4; IL-10	Probably beneficial
Gao, 2020 [60]	RCT: 22	2085	(1)(2)(3)(4)(5)(6)(9)(8)	NM	TwHFPs vs CWM + COP	JS	Yes	AEs	No effect
Ying, 2021 [61]	RCT: 13	1004	(1)(2)(3)(4)(6)(8)(9)	ACR 1987; ACR/EULAR 2009	TwHFPs + LEF vs LEF	CROB	Yes	CTE; MS; TJC; SJCAEs; CRP; ESR; RF; IgA; IgG; IgM; IL-1; IL-6; IL-10; TNF-*α*; sICAM-1	Probably beneficial
Wang, 2021 [62]	Mixed RCT and CCT: 10	696	(1)(2)(3)(6)(8)	ACR 1987; ACR/EULAR 2009	TwHFPs vs LEF	JS	Yes	TJC; SJC; PJC; ESR; CRP; CTE	Beneficial

(1) CNKI, (2) VIP, (3) WanFang, (4) CBM-disk, (5) EMBASE, (6) PubMed, (7) CCTR, (8) WOS, (9) CL, (10) MEDLINE, (11) AMED, (12) CINAH, (13) CMFD, (14) CENTRAL, (15) ScienceDirect, (16) FMRS, (17) Elsevier; RCT: randomized controlled trial, CCT: controlled clinical trial, CTE: clinical treatment efficacy, AEs: adverse events, vs: versus, LEF: leflunomide, MTX: methotrexate, SASP: sulfasalazine, 3M: 3 months, 6M: 6 months, 1M: 1 month, MS: morning stiffness, SJC: swollen joint count, TJC: tender joint count, ESR: erythrocyte sedimentation rate, CRP: C-reactive protein, RF: rheumatoid factor, IL-1: interleukin 1, IL-4: interleukin 4, IL-6: interleukin 6, IL-10: interleukin 10, TNF-*α*: tumor necrosis factor-alpha, NM: not mentioned, GS: grip strength, 15 Mwt: 15 m walking time, 15/20 Mwt: 15/20 m walking time, TS: tenderness score, PPOA: physician-rated and patient-rated overall assessments, X-RS: X-ray score, RCJ: radiological changes of joints, WDAR: withdrawal rate related to adverse reactions, JS: joint symptoms, DAS: disease activity score, CCP: cyclic citrullinated peptide, MGS: mean grip strength, AOT: analgesic onset time, SF-36: short form 36 health questionnaire, HAQ: health assessment questionnaire, TCMSJS: TCM symptom score of the number of joint swelling, PJC: painful joint count, DMARDs: disease-modifying antirheumatic drugs, TwHFPs: Tripterygium wilfordii Hook. f. preparations, NT: no therapy, CM: conventional medicine, COP: Chinese patent medicine or placebo, CROB: Cochrane Risk of Bias tool, JS: Jadad Scale, CL: Cochrane Library, Mtss: Van der Heijde modified. Note. The conclusions reported by the included SRs were classified into five categories by referring to another evidence mapping study [63]. Inconclusive: reported the results differed across or within reviews due to conflicting results or limitations of individual studies. No effect: reported that there is no difference between intervention and comparator. Harmful: reported clearly a harmful effect. Probably beneficial: did not report firm benefits despite the reported positive treatment effect. Beneficial: reported a clear beneficial effect without major concerns regarding the supporting evidence.

**Table 2:** The results of AMSTAR-2.

Study ID	Item 1	Item 2	Item 3	Item 4	Item 5	Item 6	Item 7	Item 8	Item 9	Item 10	Item 11	Item 12	Item 13	Item 14	Item 15	Item 16	Ranking of quality
Xu 2001 [38]	Y	N	N	N	N	N	N	PY	Y	N	N	N	Y	N	N	N	−−
Canter 2006 [39]	Y	N	N	Y	N	Y	Y	Y	Y	N	N	N	Y	Y	N	N	−
Jiang 2009a [40]	Y	N	N	PY	Y	Y	Y	PY	Y	N	Y	Y	Y	Y	N	N	−
Jiang 2009b [41]	Y	N	N	PY	Y	Y	Y	PY	Y	N	Y	Y	Y	Y	N	N	−
Tang 2010 [42]	Y	N	N	PY	Y	Y	N	N	N	N	Y	N	N	N	N	N	−−
Wang 2011 [43]	Y	N	N	PY	Y	Y	Y	Y	Y	N	Y	N	N	N	N	N	−
Liu 2013 [20]	Y	N	Y	PY	Y	Y	N	PY	Y	N	Y	Y	Y	Y	Y	N	−−
Wang 2014 [44]	Y	N	N	PY	Y	Y	N	Y	Y	Y	N	N	Y	N	N	Y	−−
Yang 2016 [46]	Y	N	N	N	Y	Y	Y	PY	Y	N	Y	Y	N	N	Y	Y	−
Wang 2016 [45]	Y	Y	N	Y	Y	Y	N	Y	Y	Y	Y	Y	Y	Y	Y	Y	−
Zeng 2017 [47]	Y	N	N	PY	Y	Y	N	PY	Y	N	Y	N	N	N	N	N	−−
Wang 2017 [21]	Y	Y	N	Y	Y	Y	N	Y	Y	Y	Y	Y	Y	Y	Y	Y	−
He 2018 [50]	Y	N	N	PY	Y	Y	N	PY	Y	Y	N	N	N	N	N	N	−−
Wang 2018 [48]	Y	N	N	PY	Y	Y	N	Y	Y	Y	Y	Y	Y	Y	N	Y	−−
Zhou 2018 [49]	Y	N	N	PY	Y	Y	N	Y	Y	Y	Y	Y	Y	Y	Y	N	−−
Wang 2019 [51]	Y	N	N	PY	Y	Y	N	PY	Y	Y	Y	N	N	Y	Y	N	−−
Li 2019 [52]	Y	N	N	PY	Y	Y	N	PY	Y	Y	Y	Y	Y	Y	Y	N	−−
Ying 2019 [53]	Y	N	N	PY	Y	Y	N	PY	Y	Y	N	N	N	N	Y	Y	−−
Zhu 2019 [54]	Y	N	N	PY	Y	Y	N	PY	Y	Y	N	N	N	N	N	N	−−
Chen 2020 [57]	Y	N	N	PY	Y	Y	N	PY	PY	Y	N	N	N	N	Y	N	−−
Li 2020 [58]	Y	N	N	Y	Y	Y	N	PY	PY	Y	Y	Y	Y	Y	Y	N	−−
Wang 2020 [59]	Y	N	N	PY	Y	Y	N	PY	Y	Y	Y	Y	Y	Y	Y	N	−−
Gao 2020 [60]	Y	N	N	PY	Y	Y	N	PY	Y	Y	Y	N	N	N	N	N	−−
Wen 2020 [55]	Y	N	N	Y	Y	Y	N	Y	Y	N	Y	Y	Y	Y	Y	Y	−−
Yang 2020 [56]	Y	N	N	PY	Y	Y	N	Y	PY	N	Y	Y	N	Y	Y	Y	−−
Ying 2021 [61]	Y	N	N	PY	Y	Y	N	Y	PY	Y	Y	Y	Y	Y	Y	N	−−
Wang 2021 [62]	Y	N	N	PY	Y	Y	N	PY	PY	Y	N	N	Y	Y	Y	Y	−−

*Note.* Y: yes; PY: partial yes; N: no; ++: high; +: moderate, −: low; −−: critically low.

**Table 3:** The results of GRADE.

Outcomes	Study ID	Synthesis of results	Total patient number in the treatment or control group	No. of participants (studies)	Quality of the evidence (GRADE)
SJC	Liu 2013 [20]	MD −4.13, 95% CI (−5.69, −2.58), *I*^2^ = 0%, *P* < 0.00001	45/47	2	⨁⨁◯◯LOW^a,b^
	Yang 2020 [56]	MD −1.24, 95% CI (−1.59, −0.88), *I*^2^ = 97%, *P* < 0.00001	417/417	12	⨁⨁⨁◯MODERATE^a^
	Zhou Y 2018 [49]	MD −1.92, 95% CI (−3.85, 0.03), *I*^2^ = 0%, *P* < 0.00001	219/218	6	⨁⨁◯◯LOW^a,b^
	He 2018 [50]	MD 0, 95% CI (−0.19, 0.2), *I*^2^ = 41%, *P*=1.00	30/30	3	⨁⨁◯◯LOW^a,b^
	Wang 2019 [51]	MD 3.01, 95% CI (2.09, 3.93), *I*^2^ = 88%, *P* < 0.00001	635/633	14	⨁⨁⨁◯MODERATE^a^
	Wang 2021 [62]	SMD −0.64, 95% CI (−1.32, 0.05), *I*^2^ = 93%, *P*=0.07	287/287	8	⨁⨁⨁◯MODERATE^a^
	Yang 2016 [46]	MD −1.96, 95 % CI (−3.56, 0.35), *I*^2^ = 87%, *P*=0.14	69/68	2	⨁◯◯◯VERY LOW^a,b^
	Yin 2019 [53]	SMD −1.46, 95% CI (−2.4, −0.44), *I*^2^ = 97%, *P*=0.005	342/330	8	⨁⨁⨁◯MODERATE^a^
	Yin 2021 [61]	SMD −0.78, 95% CI (−1.52, −0.04) ,*I*^2^ = 95%, *P*=0.04	362/362	10	⨁⨁⨁◯MODERATE^a^
	Wen 2020 [55]	SMD −1.72, 95% CI (−2.04, −1.41), *I*^2^ = 89%, *P*=0.0001	1196/1188	30	⨁⨁◯◯LOW^a^

MS	Yang, 2020 [56]	MD −0.29, 95% CI (−0.42, −0.12) , *I*^2^ = 99%, *P*=0.0005	296/296	8	⨁⨁⨁◯MODERATE^a^
	Zhou 2018 [49]	MD −30.94, 95% CI (−37.85, −24.04), *I*^2^ = 86%, *P*=0.21	144/142	3	⨁⨁◯◯LOW^a^
	He 2018 [50]	MD −0.32, 95% CI (−0.4, −0.24), *I*^2^ = 38%, *P*=0.0001	66/66	2	⨁⨁◯◯LOW^a^
	Wang 2019 [51]	MD −18.24, 95% CI (−12.64, 23.84), *I*^2^ = 36.9%, *P* < 0.00001	383/383	9	⨁⨁⨁◯MODERATE^a^
	Yin 2019 [53]	SMD −1.51, 95% CI (−2.31, −0.71), *I*^2^ = 94%, *P*=0.00002	267/267	6	⨁⨁◯◯LOW^a^
	Yin 2021 [61]	SMD −2.29, 95% CI (−3.36, −1.12), *I*^2^ = 0%, *P* < 0.00001	100/100	3	⨁⨁◯◯LOW^a^

RF	Liu 2013 [20]	MD −32.4, 95% CI (−89.76, −24.96), *I*^2^ = 24%, *P*=2.7	45/47	2	⨁⨁◯◯LOW^a^
	Wang 2018 [48]	MD −5.41, 95% CI (−7.46, −3.37), *I*^2^ = 13%, *P* < 0.00001	197/202	3	⨁⨁◯◯LOW^a^
	Yang 2020 [56]	MD −50.88, 95% CI (−72.3, 29.45), *I*^2^ = 99%, *P* < 0.00001	257/257	7	⨁◯◯◯VERY LOW^a,b,c^
	Jiang 2009b [41]	MD −0.5, 95% CI (−0.81, −0.18), *I*^2^ = 85.1%, *P*=0.002	85/85	3	⨁⨁◯◯LOW^a,b^
	Li 2019 [52]	SMD 1.05, 95% CI (0.51, 1.6), *I*^2^ = 94%, *P*=0.00001	521/521	12	⨁⨁⨁◯MODERATE^a^
	Wang 2011 [43]	MD 0.38, 95% CI (−0.42, 1.18), *I*^2^ = 64%, *P*=0.36	55/55	2	⨁⨁◯◯LOW^a,b^
	Wang 2021 [62]	SMD −2.23, 95% CI (−3.27, −1.19), *I*^2^ = 95%, *P* < 0.00001	234/234	7	⨁⨁⨁◯MODERATE^a^
	Yin 2019 [53]	SMD −1.11, 95% CI (−1.96, −0.26), I^2^ = 94%, *P*=0.01	215/215	5	⨁⨁◯◯LOW^a,b^
	Yin 2021 [61]	SMD −2.97, 95% CI (−4.22, −1.72), I^2^ = 97%, *P* < 0.00001	327/327	8	⨁⨁⨁◯MODERATE^a^

TJC	Yang 2020 [56]	SMD −50.88, 95% CI(−72.30, 29.45.48), *I*^2^ = 99%, *P* < 0.00001	894/893	23	⨁◯◯◯VERY LOW^a,b,c^
	Zhou 2018 [49]	MD −1.51, 95% CI (−2.2, −0.83), *I*^2^ = 0%, *P* < 0.00001	417/417	12	⨁⨁⨁◯MODERATE^a^
	Wang 2019 [51]	MD 2.15, 95% CI (−3.54, −−0.75), *I*^2^ = 78%, *P* < 0.00001	219/218	6	⨁⨁◯◯LOW^a,b^
	Wang 2021 [62]	SMD −0.92, 95% CI (−1.74, −0.09), *I*^2^ = 93%, *P*=0.03	190/190	6	⨁⨁⨁◯MODERATE^a^
	Yang 2016 [46]	MD −2.73, 95% CI (−4.68, −0.78), *I*^2^ = 0%, *P*=0.06	69/68	2	⨁◯◯◯VERY LOW^a,b,c^
	Yin 2019 [53]	SMD −1.28, 95% CI (−1.98, −0.57), *I*^2^ = 95%, *P*=0.0004	382/370	9	⨁⨁⨁◯MODERATE^a^
	Yin 2021 [61]	SMD −0.92, 95% CI (−1.74, −0.09), *I*^2^ = 93%, *P*=0.003	362/36	10	⨁⨁⨁◯MODERATE^a^
	Wen 2020 [55]	SMD −1.69, 95% CI (−2.01, −1.37), *I*^2^ = 89%, *P* ≤ 0.001	1233/1225	31	⨁⨁⨁◯MODERATE^a^

Total effective rate	Wang 2018 [48]	RR 1.20, 95% CI (1.13, 1.27), *I*^2^ = 31%, *P* < 0.00001	950/516	7	⨁⨁⨁◯MODERATE^a^
	Zeng 2017 [47]	OR 1.02, 95% CI (0.46, 2.28), *I*^2^ = 0%, *P*=0.95	103/101	4	⨁⨁◯◯LOW^a,b^
	He 2018 [50]	RR 1.19, 95% CI (1.02, 1.38), *I*^2^ = 0%, *P*=0.02	73/71	3	⨁⨁◯◯LOW^a,b^
	Wang 2021 [62]	OR 3.80, 95% CI (2.34, 6.16), *I*^2^ = 0%, *P* < 0.00001	253/253	7	⨁⨁⨁◯MODERATE^a^
	Yin 2019 [53]	RR 1.23, 95% CI (1.13, 1.35), *I*^2^ = 20%, *P* < 0.00001	296/296	7	⨁⨁◯◯LOW^a,b^
	Yin 2021 [61]	OR 4.27, 95% CI (2.51, 7.27), *I*^2^ = 0%, *P* < 0.00001	3668/36	9	⨁⨁⨁◯MODERATE^a^
	Wen 2020 [55]	RR1.23, 95% CI (1.133, 1.335), *I*^2^ = 0%, *P*=0.951	452/452	12	⨁ȁ⨁◯MODERATE^a^

^*∗*^The risk in the intervention group (and its 95% confidence interval) is based on the assumed risk in the comparison group and the relative effect of the intervention (and its 95% CI). CI: confidence interval; RR: risk ratio; OR: odds ratio; MD: mean difference. GRADE Working Group grades of evidence—HIGH quality: further research is very unlikely to change our confidence in the estimate of effect; MODERATE quality: further research is likely to have an important impact on our confidence in the estimate of effect and may change the estimate; LOW quality: further research is very likely to have an important impact on our confidence in the estimate of effect and is likely to change the estimate; VERY LOW quality: we are very uncertain about the estimate. a: downgraded due to risk of bias; b: downgraded due to publication bias; c: downgraded due to inconsistency and imprecision.

## Data Availability

The data supporting this overview of systematic reviews are from previous studies and data sets, which have been cited. The processed data are available from the corresponding author upon reasonable request.

## Abbreviations

CNKI: China National Knowledge Infrastructure
VIP: VIP database
RCT: Randomized controlled trial
CCT: Controlled clinical trial
CTE: Clinical treatment efficacy
AEs: Adverse events
VS: Versus
LEF: Leflunomide
MTX: Methotrexate
SASP: Sulfasalazine
3M: 3 months
6M: 6 months
1M: 1 month
MS: Morning stiffness
SJC: Swollen joint count
TJC: Tender joint count
ESR: Erythrocyte sedimentation rate
CRP: C-reactive protein
RF: Rheumatoid factor
IL-1: Interleukin 1
IL-4: Interleukin 4
IL-6: Interleukin 6
IL-10: Interleukin 10
TNF-*α*: Tumor necrosis factor-alpha
NM: Not mentioned
GS: Grip strength
15Mwt: 15 m walking time
15/20Mwt: 15/20 m walking time
TS: Tenderness score
PAOS: Physician and patient-rated overall assessments
X-RS: X-ray score
RCJ: Radiological changes of joints
WDAR: Withdrawal rate related to adverse reactions
JS: Joint symptoms
DAS: Disease activity score
CCP: Cyclic citrullinated peptide
MGS: Mean grip strength
AOT: Analgesic onset time
SF-36: Short form 36 health questionnaire
HAQ: Health assessment questionnaire
TCMSJS: TCM symptom score of the number of joint swelling
PJC: Painful joint count
DMARDs: Disease-modifying antirheumatic drugs
TwHF preparations: *Tripterygium wilfordii Hook. f.* preparations
NT: No therapy
CM: Conventional medicine
COP: Chinese patent medicine or placebo
CROB: Cochrane Risk of Bias tool
JS: Jadad Scale
CL: Cochrane Library.

## Supplementary Materials

Supplementary Materials Appendix A: search strategies and excluded systematic reviews. Appendix B: the results of ROBIS and PRISIMA.
