# Supplementary material for: Tripterygium wilfordii Hook. f. Preparations for Rheumatoid Arthritis: An Overview of Systematic Reviews
Source: Evid Based Complement Alternat Med. 2022 Apr 12;2022:3151936. doi: 10.1155/2022/3151936 (PMC9019410; doi:10.1155/2022/3151936)
Supplement: Supplementary Materials — Appendix A: search strategies and excluded systematic reviews. Appendix B: the results of ROBIS and PRISIMA. [file 3151936.f1.docx]

| Appendix A  Search strategies | |
| --- | --- |
| **Database** | **Search strategy** |
| CNKI database(Chinese database)  （https://www.cnki.net/） | (SU = 'lei feng shi guan jie yan ' OR SU = 'lei feng shi xing guan jie yan ' OR SU = ' RA ' OR SU = 'guan jie yan , lei feng shi ') AND (SU='xi tong ping jia ' OR SU='xi tong zong shu ' OR SU=' Meta fen xi ' OR SU='hui cui fen xi ') |
| VIP database(Chinese database)  （http://www.cqvip.com/） | (M=lei feng shi guan jie yan OR lei feng shi xing guan jie yan OR guan jie yan , lei feng shi OR RA) AND (M=xi tong ping jia OR xi tong zong shu OR Meta fen xi OR hui cui fen xi ) |
| Wan Fang database(Chinese database)  （https://www.wanfangdata.com.cn/index.html） | ti ming huo guan jian ci :(("lei feng shi guan jie yan " OR "lei feng shi xing guan jie yan " OR "guan jie yan , lei feng shi " OR "RA") AND ("xi tong ping jia " OR "xi tong zong shu " OR "Meta fen xi " OR "hui cui fen xi ")) |
| CBM database(Chinese database)  （http://www.sinomed.ac.cn/） | #1 "xi tong ping jia "[chang yong zi duan :zhi neng ] OR "xi tong zong shu "[chang yong zi duan :zhi neng ] OR "Metafen xi "[chang yong zi duan :zhi neng ] OR "hui cui fen xi "[chang yong zi duan :zhi neng ]  #2 "lei feng shi guan jie yan "[chang yong zi duan :zhi neng ] OR "lei feng shi xing guan jie yan "[chang yong zi duan :zhi neng ] OR "RA"[chang yong zi duan :zhi neng ] OR "guan jie yan ,"[chang yong zi duan :zhi neng ] AND "lei feng shi "[chang yong zi duan :zhi neng ]  #3 (#2) AND (#1) |
| Pubmed  （https://pubmed.ncbi.nlm.nih.gov/） | Search:(("Systematic Review" [Publication Type]) AND (("Arthritis, Rheumatoid"[Mesh]) OR ((Arthritis, Rheumatoid[Title/Abstract]) OR (Rheumatoid Arthritis[Title/Abstract])))) AND ((((((((((((((((Medicine, Chinese Traditional[Title/Abstract]) OR (Traditional Chinese Medicine[Title/Abstract])) OR (Chung I Hsueh[Title/Abstract])) OR (Hsueh, Chung I[Title/Abstract])) OR (Traditional Medicine, Chinese[Title/Abstract])) OR (Zhong Yi Xue[Title/Abstract])) OR (Chinese Traditional Medicine[Title/Abstract])) OR (Chinese Medicine, Traditional[Title/Abstract])) OR (Traditional Tongue Diagnosis[Title/Abstract])) OR (Tongue Diagnoses, Traditional[Title/Abstract])) OR (Tongue Diagnosis, Traditional[Title/Abstract])) OR (Traditional Tongue Diagnoses[Title/Abstract])) OR (Traditional Tongue Assessment[Title/Abstract])) OR (Tongue Assessment, Traditional[Title/Abstract])) OR (Traditional Tongue Assessments[Title/Abstract])) OR ("Medicine, Chinese Traditional"[Mesh])) |
| EMBASE  (https://www.elsevier.com/solutions/embase-biomedical-research) | #1‘arthritis deformans’ab,ti OR ‘arthritis’’ab,ti OR ‘rheumatoid’ab,ti OR‘arthrosis deformans’ab,ti OR‘ beauvais disease’ab,ti OR‘chronic articular rheumatism’ab,ti OR‘chronic polyarthritis’ab,ti OR ‘chronic progressive poly arthritis’ab,ti OR‘chronic progressive polyarthritis’ab,ti OR‘chronic rheumatoid arthritis’ab,ti OR‘disease, beauvais’ab,ti OR‘infantile rheumatoid arthritis’ab,ti OR‘ inflammatory arthritis’ab,ti OR‘ polyarthritis, primary chronic’ab,ti OR‘ primary chronic polyarthritis’ab,ti OR‘ rheumarthritis’ab,ti OR ‘rheumatic arthritis’ab,ti OR‘rheumatic polyarthritis’ab,ti OR‘rheumatism, chronic articular’ab,ti  #2‘Chinese herbal medicine’ab,ti OR ‘medicine, Chinese traditional’ab,ti OR ‘ traditional Chinese medicine’ab,ti  #3 “systematic review”  #4 #1 AND #2 AND #3 |
| Cochrane  (https://www.cochranelibrary.com) | #1MeSH descriptor: [Arthritis, Rheumatoid] explode all trees  #2MeSH descriptor: [Medicine, Chinese Traditional] explode all trees  #3#1 and #2  #4(rheumatoid arthritis):ti,ab,kw OR (arthritis deformans):ti,ab,kw OR (arthritis, rheumatoid):ti,ab,kw OR (arthrosis deformans):ti,ab,kw OR (beauvais disease):ti,ab,kw  (Word variations have been searched)S Limits  #5(chronic articular rheumatism):ti,ab,kw OR (chronic polyarthritis):ti,ab,kw OR (chronic progressive poly arthritis):ti,ab,kw OR (chronic progressive polyarthritis):ti,ab,kw OR (chronic rheumatoid arthritis):ti,ab,kw  #6(disease, beauvais):ti,ab,kw OR (infantile rheumatoid arthritis):ti,ab,kw OR (inflammatory arthritis):ti,ab,kw OR (polyarthritis, primary chronic):ti,ab,kw OR (primary chronic polyarthritis):ti,ab,kw  #7(rheumarthritis):ti,ab,kw OR (rheumatic arthritis):ti,ab,kw OR (rheumatic polyarthritis):ti,ab,kw OR (rheumatism, chronic articular):ti,ab,kw OR (Rheumatoid Arthritis):ti,ab,kw  #8 #4 OR #5 OR #6 OR #7 OR #1  #9(Chinese Traditional Medicine):ti,ab,kw OR (Chinese Medicine, Traditional):ti,ab,kw OR (Traditional Tongue Diagnosis):ti,ab,kw OR (Traditional Tongue Assessment):ti,ab,kw OR (Tongue Assessment, Traditional):ti,ab,kw  #10(Traditional Tongue Assessments):ti,ab,kw(Word variations have been searched)S  #11 #9 OR #10 OR #2  #12 #8 AND #11 |

Excluded systematic reviews

| Record | Excluded reasons |
| --- | --- |
| The Effectiveness and Safety of Tripterygium wilfordii Hook. F Extracts in Rheumatoid Arthritis: A Systematic Review and Meta-Analysis. Frontiers in Pharmacology, 2018. 9. | duplicated |
| Luo, J., et al., Benefits and Safety of Tripterygium Glycosides and Total Glucosides of Paeony for Rheumatoid Arthritis: An Overview of Systematic Reviews. Chin J Integr Med, 2019. 25(9): p. 696-703. | Not a systematic review |
| Zhu Gz.et al., Systematic evaluation and correlation between CYP3A4 gene polymorphism and curative effect of Tripterygium wilfordii on rheumatoid arthritis [D] Beijing University of traditional Chinese medicine, 2020 | the population of the SR was not RA patients |
| Yang J et al., Systematic evaluation of the effect of Tripterygium wilfordii polyglycoside tablets on pro-inflammatory cytokines in rheumatoid arthritis Chinese Journal of traditional Chinese medicine, 2020 45 (04): page 764-774 | the population of the SR was not RA patients |
| Sun PP, et al. Systematic evaluation of the distribution characteristics of clinical adverse reactions of Tripterygium Wilfordii and its preparations World science and technology - modernization of traditional Chinese medicine, 2015 17 (09): 1899-1905 | the population of the SR was not RA patients |
| Sun PP, et al. Systematic evaluation and meta analysis of randomized controlled trials on the distribution characteristics of clinical adverse reaction events of Tripterygium Wilfordii and its preparations, 2015, Beijing University of traditional Chinese medicine | the population of the SR was not RA patients |

Appendix B

TABLE 1: The Results of ROBIS.

| Study ID | Phase 1  Assessing relevance | Phase 2 | | | | Phase 3  Risk of bias in the review |
| --- | --- | --- | --- | --- | --- | --- |
|  |  | 1. Study eligibility criteria | 2. Identification and selection of studies | 3. Data collection and study appraisal | 4. Synthesis and findings |  |
| Xu 2001 | Low | High | High | High | High | High |
| Canter 2006 | Low | High | High | High | Low | Low |
| Jiang 2009a | Low | High | Low | Low | High | High |
| Jiang 2009b | Low | High | Low | Low | High | High |
| Tang 2010 | Low | High | High | High | High | Low |
| Wang 2011 | Low | High | Low | Low | High | High |
| Liu 2013 | Low | High | Low | Low | Low | High |
| Wang 2014 | Low | High | Low | High | High | High |
| Yang 2016 | Low | High | High | High | High | High |
| Wang2016 | Low | LOW | High | Low | Low | Low |
| Zeng 2017 | Low | High | High | Low | High | High |
| Wang2017 | Low | High | Low | Low | Low | High |
| He 2018 | Low | High | High | High | High | Low |
| Wang2018 | Low | High | Low | High | Low | High |
| Zhou2018 | Low | High | High | Low | Low | Low |
| Wang2019 | Low | High | High | Low | Low | High |
| Li2019 | Low | High | High | Low | High | High |
| Ying2019 | Low | High | High | Low | Low | High |
| Zhu2019 | Low | High | Low | High | Low | High |
| Chen2020 | Low | High | High | Low | Low | High |
| Li2020 | Low | High | High | Low | Low | High |
| Wang2020 | Low | High | Low | High | Low | High |
| Gao2020 | Low | High | High | High | Low | Low |
| Wen 2020 | Low | High | High | Low | Low | High |
| Yang 2020 | Low | High | High | Low | High | Low |
| Ying2021 | Low | High | High | Low | Low | High |
| Wang2021 | Low | High | High | High | Low | High |

NOTE: Low=low risk; High= high

TABLE 2 The Results of PRISIMA.

|  | **Items** | | | | | | | | | | | | | | | | | | | | | | | | | | |
| --- | --- | --- | --- | --- | --- | --- | --- | --- | --- | --- | --- | --- | --- | --- | --- | --- | --- | --- | --- | --- | --- | --- | --- | --- | --- | --- | --- |
| **Study ID** | **Q1** | **Q2** | **Q3** | **Q4** | **Q5** | **Q6** | **Q7** | **Q8** | **Q9** | **Q10** | **Q11** | **Q12** | **Q13** | **Q14** | **Q15** | **Q16** | **Q17** | **Q18** | **Q19** | **Q20** | **Q21** | **Q22** | **Q23** | **Q24** | **Q25** | **Q26** | **Q27** |
| Xu 2001 | N | N | Y | Y | Y | Y | N | N | N | Y | Y | Y | Y | N | N | Y | Y | Y | Y | Y | N | N | Y | N | N | N | N |
| Canter 2006 | Y | N | Y | Y | Y | Y | N | N | Y | Y | Y | PY | PY | N | N | Y | Y | Y | Y | Y | N | N | N | N | N | N | N |
| Jiang 2009a | Y | N | Y | Y | Y | Y | N | Y | Y | Y | Y | Y | Y | N | N | Y | Y | Y | Y | Y | N | N | Y | N | N | N | N |
| Jiang 2009b | Y | N | Y | Y | Y | Y | N | Y | Y | Y | Y | Y | Y | N | N | Y | Y | Y | Y | Y | N | N | Y | N | N | N | N |
| Tang 2010 | N | N | Y | Y | Y | Y | N | Y | Y | Y | N | Y | Y | N | N | Y | Y | N | Y | Y | N | N | Y | N | N | N | N |
| Wang 2011 | Y | N | Y | Y | Y | Y | Y | Y | Y | Y | Y | Y | Y | N | N | Y | Y | Y | Y | Y | N | N | Y | N | N | N | N |
| Liu 2013 | Y | N | Y | Y | Y | Y | N | Y | Y | Y | Y | Y | Y | Y | N | Y | Y | N | Y | Y | Y | N | Y | N | N | N | Y |
| Wang 2014 | N | N | Y | Y | Y | Y | N | Y | Y | Y | Y | Y | N | N | Y | Y | Y | Y | Y | Y | N | N | Y | N | Y | N | N |
| Yang 2016 | N | N | Y | Y | Y | Y | N | Y | Y | Y | Y | Y | N | N | Y | Y | Y | Y | Y | Y | N | N | Y | N | N | N | N |
| Wang2016 | Y | Y | Y | Y | Y | Y | N | Y | Y | Y | Y | Y | Y | N | Y | Y | Y | Y | Y | Y | Y | N | Y | Y | Y | Y | Y |
| Zeng 2017 | N | N | Y | Y | Y | Y | N | Y | Y | Y | Y | Y | N | N | Y | Y | N | Y | Y | Y | N | N | Y | N | N | N | N |
| Wang2017 | Y | N | Y | Y | Y | Y | N | Y | Y | Y | Y | Y | Y | N | Y | Y | Y | Y | Y | Y | Y | N | Y | N | Y | Y | Y |
| He 2018 | Y | N | Y | Y | Y | Y | N | Y | Y | Y | Y | Y | N | N | Y | Y | Y | Y | Y | Y | N | N | Y | N | Y | N | N |
| Wang2018 | Y | N | Y | Y | Y | Y | N | Y | Y | Y | Y | Y | N | N | Y | Y | Y | Y | Y | Y | N | N | Y | N | Y | Y | Y |
| Zhou2018 | Y | N | Y | Y | Y | Y | N | Y | Y | Y | Y | Y | Y | Y | N | Y | Y | Y | Y | Y | Y | N | Y | N | Y | Y | N |
| Wang2019 | N | N | Y | Y | Y | Y | N | Y | Y | Y | Y | Y | Y | Y | N | Y | Y | Y | Y | Y | Y | N | Y | N | Y | N | Y |
| Li2019 | N | N | Y | Y | Y | Y | N | Y | Y | Y | Y | Y | Y | Y | N | Y | Y | Y | Y | Y | Y | N | Y | N | Y | N | N |
| Ying2019 | N | N | Y | Y | Y | Y | N | Y | Y | Y | Y | Y | Y | Y | N | Y | Y | Y | Y | Y | Y | N | Y | N | Y | Y | N |
| Zhu2019 | Y | N | Y | Y | Y | Y | N | Y | Y | Y | Y | Y | Y | N | N | Y | Y | Y | Y | Y | N | N | Y | N | Y | N | N |
| Chen2020 | N | N | Y | Y | Y | Y | N | Y | Y | Y | Y | Y | Y | Y | N | Y | Y | Y | Y | Y | Y | N | Y | N | Y | N | N |
| Li2020 | Y | N | Y | Y | Y | Y | N | Y | Y | Y | Y | Y | Y | N | Y | Y | Y | Y | Y | Y | Y | N | Y | N | Y | N | N |
| Wang2020 | Y | N | Y | Y | Y | Y | N | Y | Y | Y | Y | Y | N | N | Y | Y | Y | N | Y | Y | N | N | Y | N | Y | N | N |
| Gao2020 | N | N | Y | Y | Y | Y | N | Y | Y | Y | Y | Y | N | N | Y | Y | Y | Y | Y | Y | N | N | Y | N | Y | N | N |
| Wen 2020 | Y | N | Y | Y | Y | Y | N | Y | Y | Y | Y | Y | Y | Y | Y | Y | Y | Y | Y | Y | Y | Y | Y | N | N | Y | Y |
| Yang 2020 | Y | N | Y | Y | Y | Y | N | Y | Y | Y | Y | Y | Y | N | Y | Y | Y | Y | Y | Y | Y | N | Y | N | N | Y | Y |
| Ying2021 | Y | N | Y | Y | Y | Y | N | Y | Y | Y | Y | Y | Y | N | Y | Y | Y | Y | Y | Y | Y | N | Y | N | Y | N | N |
| Wang2021 | N | N | Y | Y | Y | Y | N | Y | Y | Y | Y | Y | N | N | Y | Y | Y | Y | Y | Y | N | N | Y | N | Y | N | N |
| Compliance (%) | 59% | 3% | 100% | 100% | 100% | 100% | 3% | 92% | 96% | 100% | 96% | 96% | 59% | 25% | 51% | 100% | 96% | 88% | 100% | 100% | 44% | 3% | 96% | 3% | 59% | 25% | 25% |

NOTE: Y=Yes; N= No; PY=Probably Y
